# Downregulation of microRNA-31 inhibits proliferation and induces apoptosis by targeting *HIF1AN* in human keloid

**DOI:** 10.18632/oncotarget.20284

**Published:** 2017-08-16

**Authors:** Juan Zhang, Dan Xu, Na Li, Yan Li, Yongjing He, Xingbo Hu, Lechun Lyu, Li He

**Affiliations:** ^1^ Department of Dermatology, the First Affiliated Hospital of Kunming Medical University, Kunming, China; ^2^ Department of Physiology, Kunming Medical University, Kunming, China; ^3^ Department of Plastic Surgery, Second Affiliated Hospital of Kunming Medical University, Kunming, China; ^4^ Department of Orthopedics, the First People’s Hospital of Kunming, Kunming, China

**Keywords:** keloid, microRNA-31, proliferation, apoptosis, cell cycle

## Abstract

microRNAs (miRNAs) play a pivotal role in the regulation of cell proliferation and apoptosis in keloid scarring. Integrative analysis of the previous miRNA microarray revealed miRNA-31 was among the most frequently altered miRNAs in keloid and hypertrophic scar. Using qRT-PCR, we further validated miRNA-31 was increased in keloid tissues and keloid-derived fibroblasts. Moreover, downregulation of miRNA-31 inhibited the cell proliferation, induced the cell apoptosis and disturbed the cell cycle progression by targeting *HIF1AN*, a negative modulator of hypoxia inducible factor 1. Through the luciferase reporter assay, *HIF1AN* was confirmed to be a target of miRNA-31. Further studies demonstrated that miRNA-31 regulated proliferation, apoptosis and cell cycle of keloid-derived fibroblasts by mediating HIF1AN/VEGF signaling pathway. Overall, our findings shed new light on miRNA-31 as a promising therapeutic target in keloid scarring.

## INTRODUCTION

Keloid is a common dermal fibro-proliferative disease unique to human. This disease possesses some features of tumor and commonly occurs after skin injury, characterized by the excessive deposition of extracellular matrix [1]. Skin lesion is prone to formating on specific areas of the body, such as chest, shoulders, neck, ears. This disease can occur in all races, however it is more prevalent in dark-skined individuals (African American, Asian, Hispanic), with a higher incidence in females [2]. It is difficult to distinguish keloid from hypertrophic scarring in the early stage, but the former usually has the characteristics of pruritic and painful feelings, invasively growing beyond the original wound boundary, sustained growth and rarely regressing spontaneously with time, high recurrence by surgical excision [2, 3]. The etiopathogenesis of keloid formation remains unclear. Keloid can occur sporadically or in familial aggregates, both genetic and external factors (such as trauma) play significant roles in its formation [4]. Therefore, recent studies on keloid formation mechanisms have gradually shifted away from genetics to epigenetics, which include DNA methylation, histone modification and the role of non-coding RNAs, such as microRNAs (miRNAs) [5].

miRNAs are a class of endogenous, small, non-coding, single-stranded RNA molecules, with the length of about 22 nucleotides. MiRNAs can bind with the 3’-untranslated region of the target genes and inhibit the expression of the corresponding genes by degradating mRNA or inhibiting the translation of mRNA. They play important roles in various physiological and pathological processes [6]. Recent studies have demonstrated that some of the miRNAs could play pivotal roles in keloid scarring by regulating fibroblast proliferation, apoptosis and epithelial-mesenchymal transition (EMT) [1, 7, 8].

So far, several studies have been carried out to screen the differential miRNA expression profiles in skin tissue, isolated fibroblasts and serum of keloid [7, 9–14]. However, the results of different studies are not consistent. In this study, we aimed to identify the miRNA expression profiles in keloid and investigate the biological functions of miRNA-31 that may serve as a novel target for prevention and treatment in keloid scarring.

## RESULTS

### The expression of miRNA-31 was upregulated in keloid

**Figure 1:**
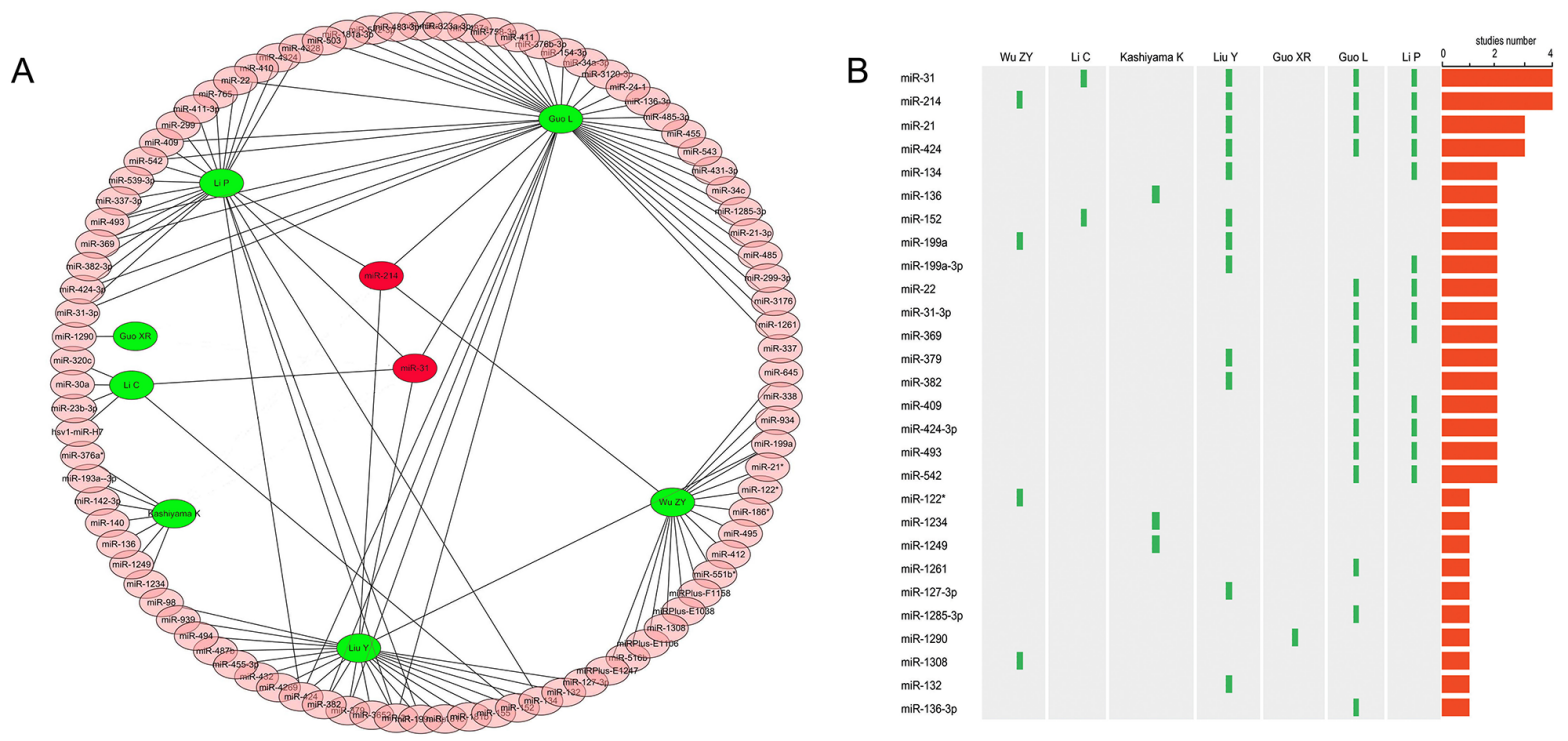
Combined analysis of previous miRNAs microarray studies **(A)**, 93 up-regulated miRNAs in seven studies; **(B)**, frequency of different miRNAs in various studies.

In total, 12 studies were initially retrieved, of which 2 studies with overlapping data, 5 studies that did not meet the inclusion criteria were excluded, leaving 7 studies that were used to perform subsequent microRNA screening of microarray data [7, 12–17]. In the included studies, 5 studies focused on keloid, and the other two studies on hypertrophic scar, involving a total of 141 different miRNAs. In 93 up-regulated miRNAs, 17 significant miRNAs were reported in at least 2 studies. Interestingly, two miRNAs, including miRNA-31 and miRNA-214, were detected by 4 studies, indicating that the both miRNAs are the common regulation factor of fibrotic disease, and may play an important role in keloid scarring (Figure 1A, 1B).

**Figure 2:**
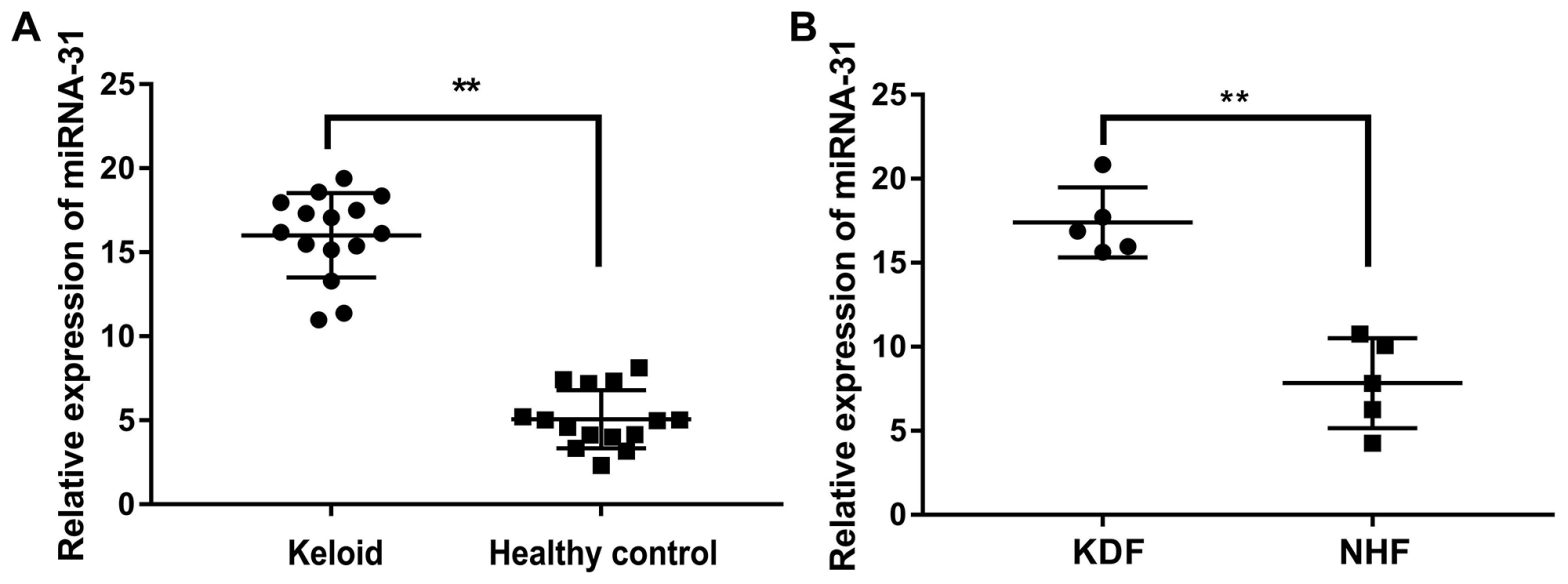
The expression of miRNA-31 in tissues and cells **(A)**, keloid tissue and healthy control tissue; **(B)**, keloid-derived fibroblasts (KDF) and normal human fibroblasts (NHF).

The expression of miRNA-31 was further validated by qRT-PCR in the skin biopsy samples of 15 keloid patients and 15 healthy controls. The results showed that the level of miRNA-31 was increased more than 3 folds (Figure 2A). In addition, we also explored the expression of miRNA-31 in keloid-derived fibroblasts and normal human fibroblasts, which was consistent with our combined analysis of previous microarray results (Figure 2B).

### Bioinformatics analysis and functional prediction of miRNA-31

TargetScan, PicTar and miRanda were used to predict the target genes of miRNA-31. The result showed there were 114 predicted target genes of miRNA-31 in all of the three programs.

**Figure 3:**
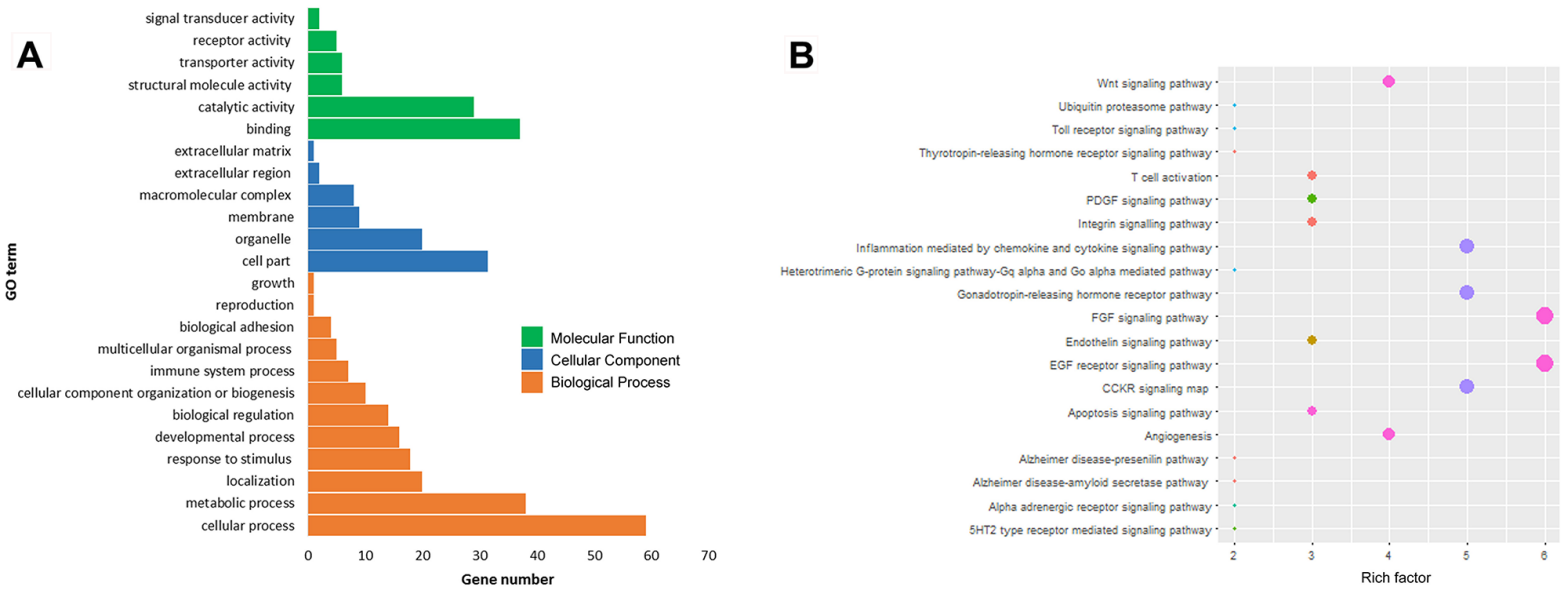
Bioinformatic analysis and functional prediction of miRNA-31 **(A)**, GO analysis of miRNA-31 target genes; **(B)**, KEGG pathway analysis.

According to GO and KEGG pathway analysis using DAVID, most of these target genes were enriched in the biological process of binding, cellular process, regulation of cell part. They were mainly related to a series of biological processes, such as FGF signaling pathway, EGF receptor signaling pathway, angiogenesis and Wnt signaling pathway (Figure 3A, 3B).

### Downregulation of miRNA-31 decreases proliferation, induces apoptosis and inhibits cell cycle of keloid-derived fibroblasts

**Figure 4:**
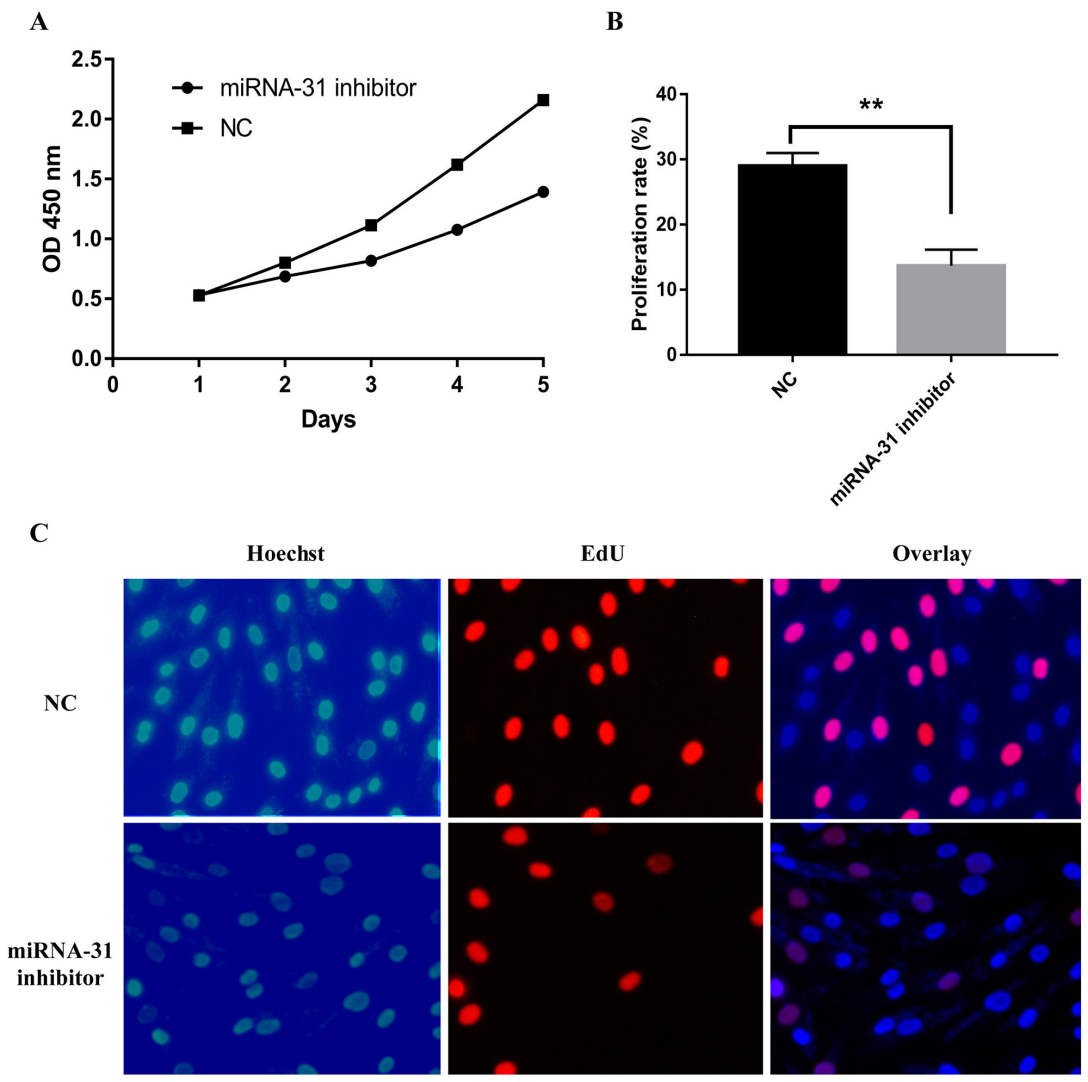
Effect of miRNA-31 on cell proliferation ability of keloid-derived fibroblasts using **(A),** CCK8 assay; **(B,C),** EdU assay. NC, negative control.

miR-31 inhibitor and relevant negative control were transfected into keloid-derived fibroblasts. The proliferation of fibroblasts was measured by CCK -8 assay. The result showed miR-31 inhibitor significantly inhibited fibroblasts vitality than negative control (Figure 4A). The effect of miR-31 on S phase of cell cycle progression was verified using EdU assay (Figure 4B, 4C). EdU staining demonstrated that miR-31 inhibitor treatment significantly suppressed DNA synthesis of fibroblasts than negative control treatment, and inhibited proliferation of fibroblasts, as demonstrated by CCK-8 assay.

**Figure 5:**
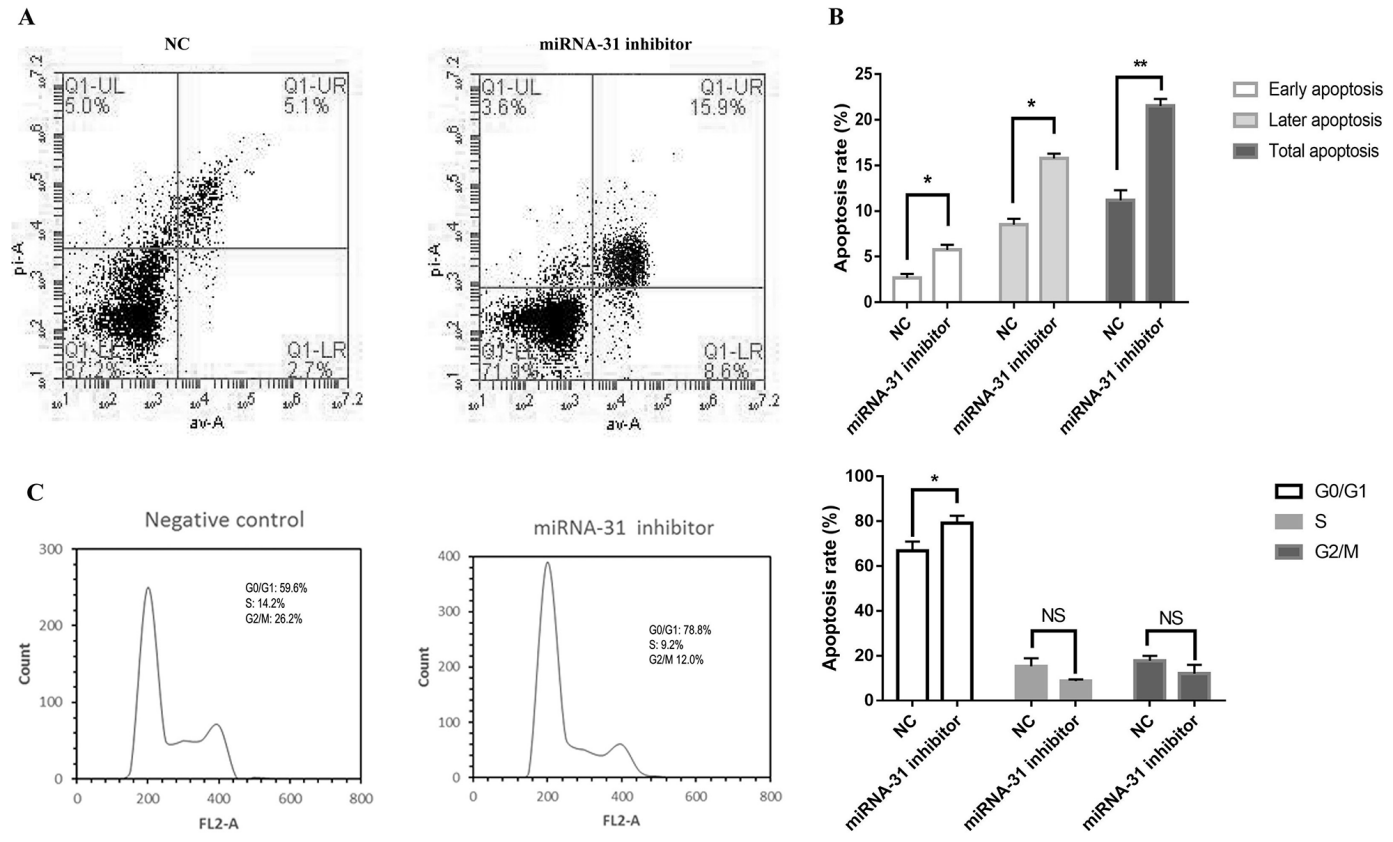
Effect of miRNA-31 on **(A, B),** apoptosis and **(C),** cell cycle of fibroblasts. NC, negative control.

Cell apoptosis and cell cycle were detected by flow cytometer. The apoptosis rate was upregulated when fibroblasts were transfected with miRNA-31 inhibitor (Figure 4A, 4B). The results of cell cycle demonstrated that the cellular proportion in G0/G1 phase in miRNA-31 inhibitor group was increased to 78.8% (Figure 5C, 5D).

### *HIF1AN* is the target gene of miRNA-31 in keloid-derived fibroblasts

**Figure 6:**
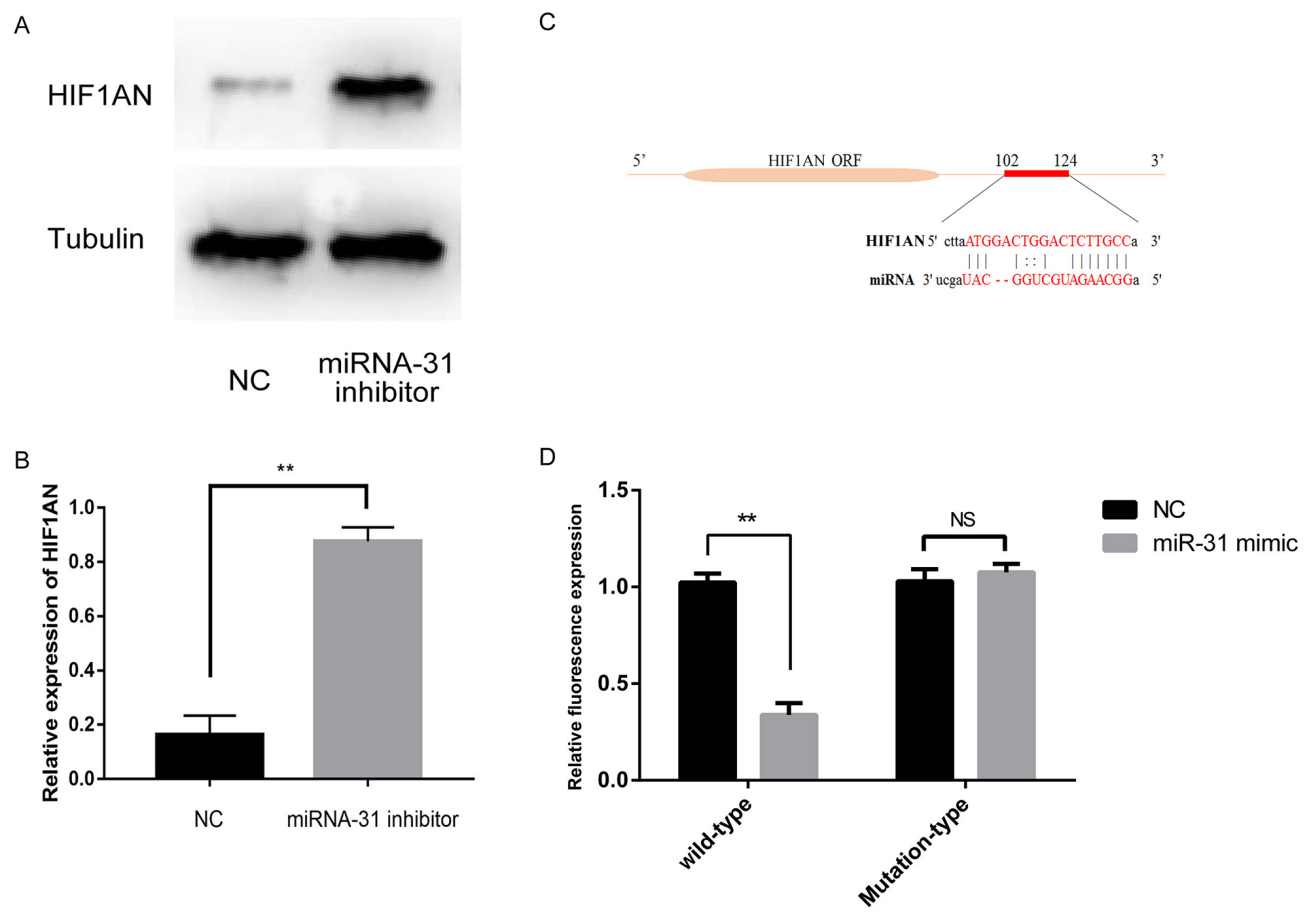
**(A, B),** The expression levels of HIF1AN were examined by Western blotting after the downregulation of miRNA-31; **(C),** miRNA-31 binding sites within *HIF1AN* 3’UTR; **(D),** Luciferase activity assays with the wild-type 3’UTR or mutated 3’UTR of *HIF1AN* cotransfeced with vector or miRNA-31. The luciferase values were normalized to the firefly luciferase activity and plotted as relative luciferase activity. NC, negative control.

Through the query in the TARGETSCAN database (http://www.targetscan.org, Version 7.1), we predicted that there was a consequential pairing between miRNA-31 and binding sites in the 3’ UTR of the gene *HIF1AN* (Figure 6C). Luciferase reporter assays were performed to validate whether *HIF1AN* expression was indeed a direct target of miRNA-31. Results showed that miRNA-31 inhibited luciferase activity in fibroblasts with the wild-type *HIF1AN* 3’-UTR reporter plasmid carried (Figure 6D). Indicating that miRNA-31 directly binds to *HIF1AN* 3’-UTR at the predicted binding sites.

In order to investigate whether miRNA-31 can affect the expression of *HIF1AN*, we extracted the total protein of fibroblasts and carried out the Western blotting. The results demonstrated that the expression level of HIF1AN increased significantly after the transfection of miRNA -31 inhibitor (Figure 6A, 6B). At the same time, we noted that the expression of miRNA-31 was negatively correlated with the protein level of HIF1AN. These results indicated that *HIF1AN* was a direct target gene of miRNA-31.

### HIF1AN is crucial to cell proliferation, apoptosis and cell cycle of keloid-derived fibroblasts

**Figure 7:**
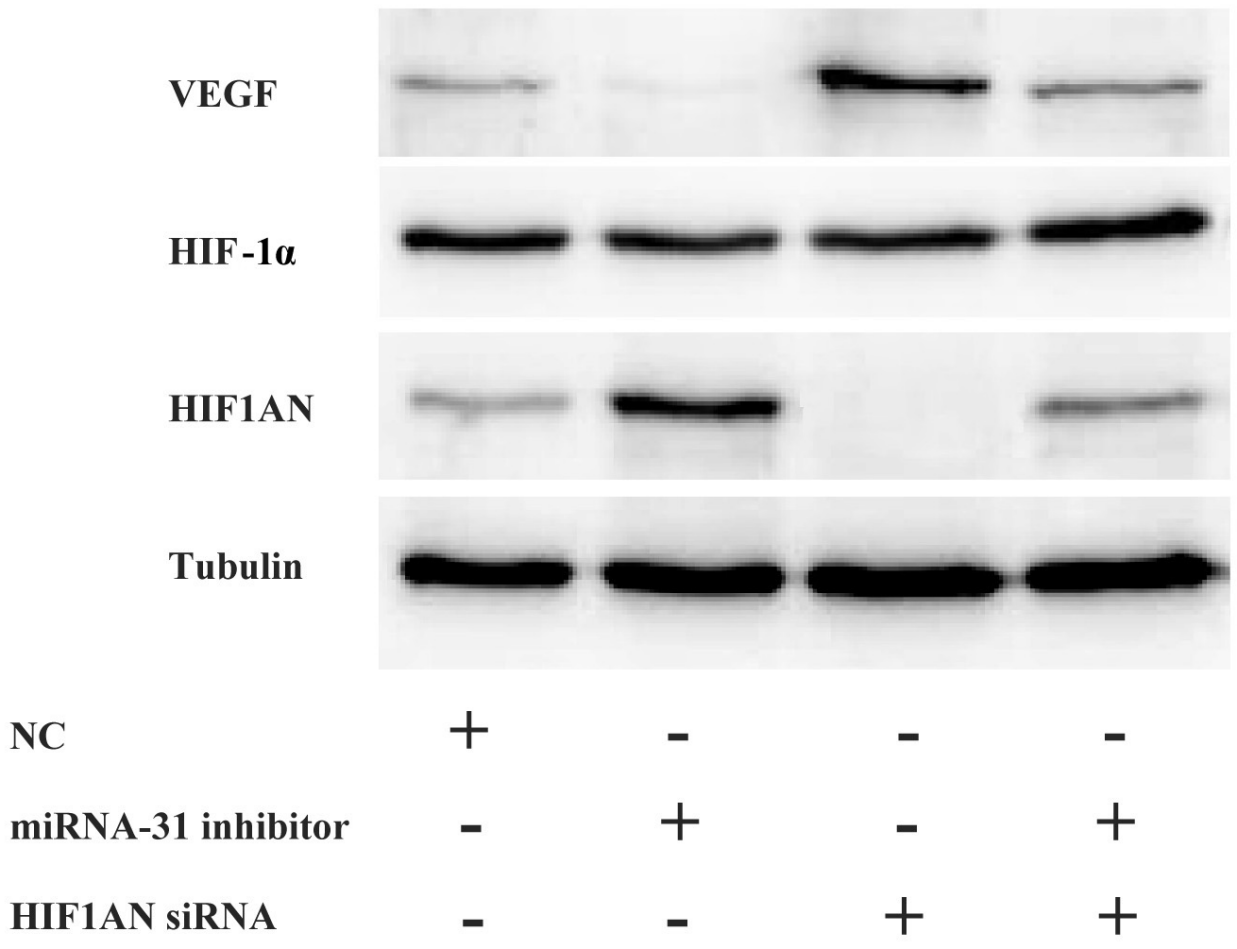
The expression levels of HIF1AN, HIF-1α and VEGF were examined by Western blotting after the downregulation of miRNA-31 and HIF1AN NC, negative control.

To explore the possible signaling pathways involving miRNA-31 and *HIF1AN* in keloid-derived fibroblasts, HIF-1α and VEGF expression level were detected. Compared to negative control group, the expression level of protein HIF1AN decreased in fibroblasts treated with specific *HIF1AN* siRNA. Meanwhile, co-treatment with *HIF1AN* siRNA and miRNA-31 inhibitor significantly downregulated the level of HIF1AN than miRNA-31 inhibitor treated cells, while the expression level of protein VEGF increased (Figure 7). However, we could not find the similar changing trend of HIF-1α.

**Figure 8:**
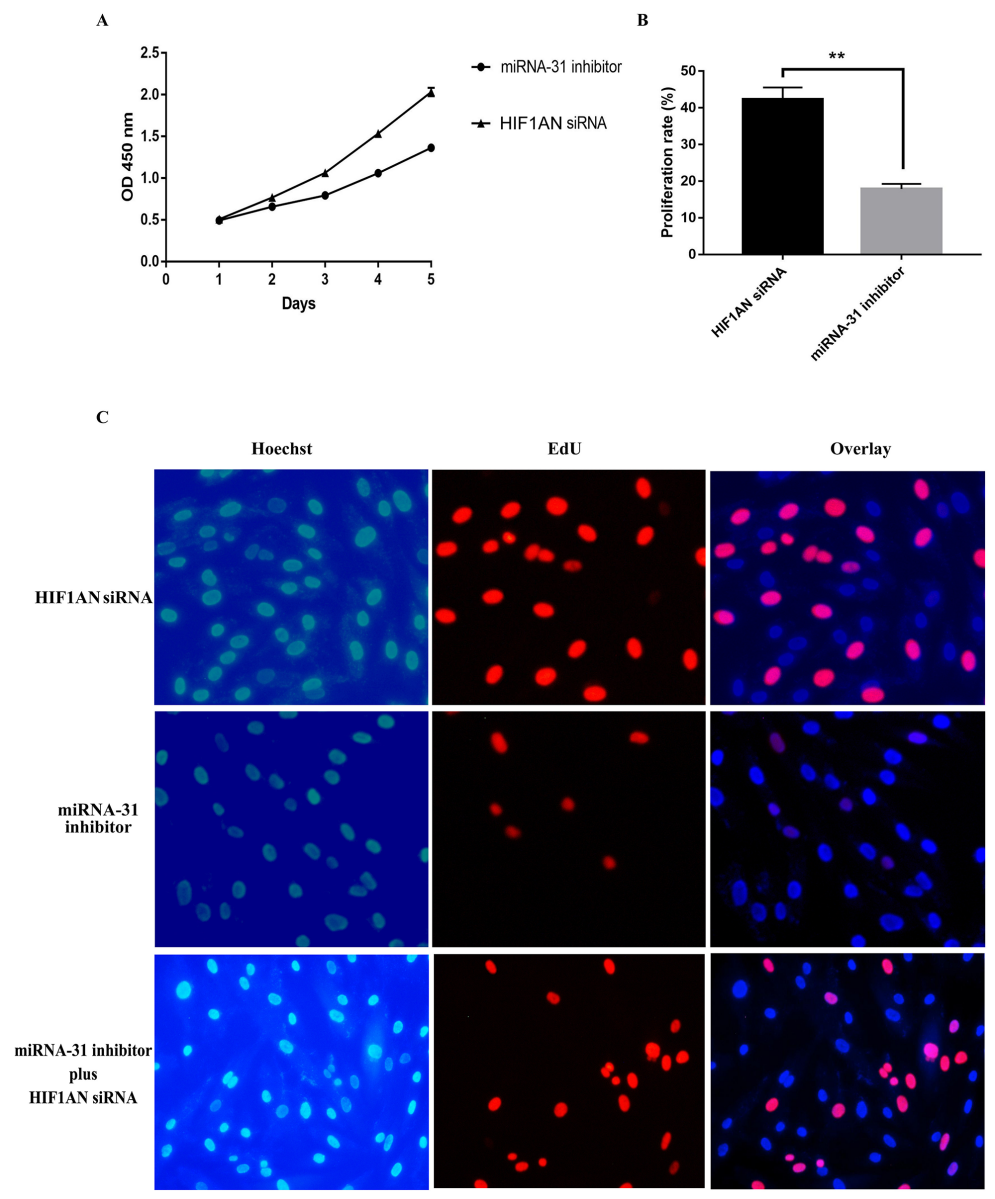
Effects of miRNA-31 siRNA and HIF1AN siRNA on cell proliferation ability of fibroblasts using **(A),** CCK8 assay; **(B,C),** EdU assay.

**Figure 9:**
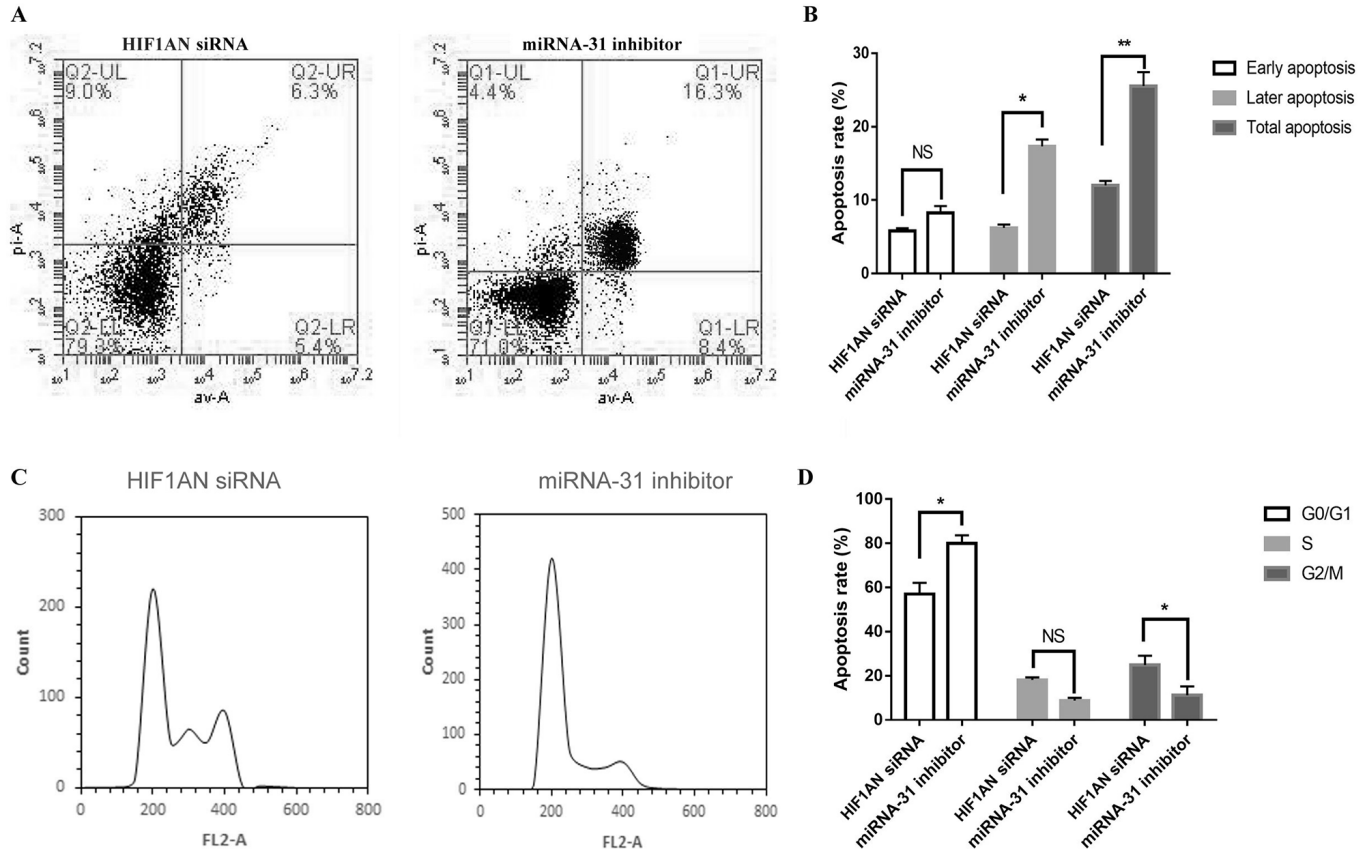
Effects of miRNA-31 siRNA and HIF1AN siRNA on **(A, B),** apoptosis and **(C, D),** cell cycle of fibroblasts.

We then explored the effect of *HIF1AN* knockdown on cell proliferation, cell apoptosis and cell cycle progression. *HIF1AN* siRNA significantly induced the cell proliferation ability, inhibited cell apoptosis and blocked fewer cell in S-phase of cell cycle (Figure 8, 9). These results suggested that miRNA-31 may regulate the proliferation, apoptosis and cell cycle of keloid-derived fibroblasts via HIF-1α/VEGF signaling pathway by targeting *HIF1AN.*

## DISCUSSION

Keloid is abnormal process of fibroblast proliferation and collagen formation after skin injury. Previous studies have demonstrated that many miRNAs were differentially expressed in the skin tissues of keloid and regulated pathologic process involved in skin fibrosis [18]. Liu Y *et al.* explored the miRNA expression profiles in tissues, fibroblasts and serum of keloid using miRNA microarray [9–11]. They showed some miRNAs were significantly altered compared with the controls and these miRNAs were demonstrated to be involved in relevant signaling pathways important for keloid scarring. miRNA-21, miRNA-181a were up-regulated in keloid and these miRNAs may negatively regulate *PTEN* and *PHLPP2* expression at the post-transcriptional level [7, 18]. Several studies also found miRNA-205, miRNA-200b, miRNA-199a, miRNA-196a and miRNA-29 were lower in keloid, which regulated the cell proliferation, apoptosis, collagen excessive deposition in human keloid fibroblasts via targeting vascular endothelial growth factor (VEGF), collagen I and collagen III [10, 12, 14, 19, 20]. Therefore, these miRNAs have been considered as potential biological markers and treatment tools in keloid scarring.

On the basis of previous miRNA microarray studies, we screened another miRNA, miRNA-31, that may have important regulatory potential in keloid. However, so far, the possible roles of miRNA-31 in this pathogenic process are still unclear. A large number of studies have demonstrated this common miRNA may be associated with skin cancers, psoriasis and autoimmune diseases. miRNA-31 has been found to be dramatically upregulated in psoriatic lesions. Further studies demonstrated that miRNA-31 was related to keratinocytes proliferation and differentiation, miRNA-31 transcription could be induced by NF-kB and thus promoted cell proliferation via inhibiting protein phosphatase 6 (ppp6c) [21]. *HIF1AN* which is inversely associated with Notch activity could be suppressed by miRNA-31 thus promoting cell differentiation. Inflammatory cytokines can promote miRNA-31 transcription, and miRNA-31 participates in psoriasis inflammation through affecting the secretion of inflammatory cytokines by targeting serine/threonine kinase 40 (STK40) which can negatively regulate NF-kB pathway [22]. Wang *et al.* found that miRNA-31 was also overexpressed in cutaneous squamous cell carcinoma (cSCC) and promoting effects of miRNA-31 in cell migration, invasion and colony formation of cSCC indicated the oncogenic role of miRNA-31 in cSCC [23]. However, there are contrary expression and functions of miRNA-31 in other tumors [24]. For example, Asangani *et al.* found miRNA-31 was downregulated in melanoma which perhaps act as an outcome of DNA methylation and histone methylation mediated by *EZH2*; miRNA-31 could inhibit the migration and invasion of melanoma cells, it might play a tumor suppressor role by inhibiting oncogenes *SRC, NIK, RAB27a, MET* [25]. In the present study, we demonstrated that miRNA-31 functions in the development of keloid scarring by negative regulation of the major components in the cell proliferation and cell apoptosis.

**Figure 10:**
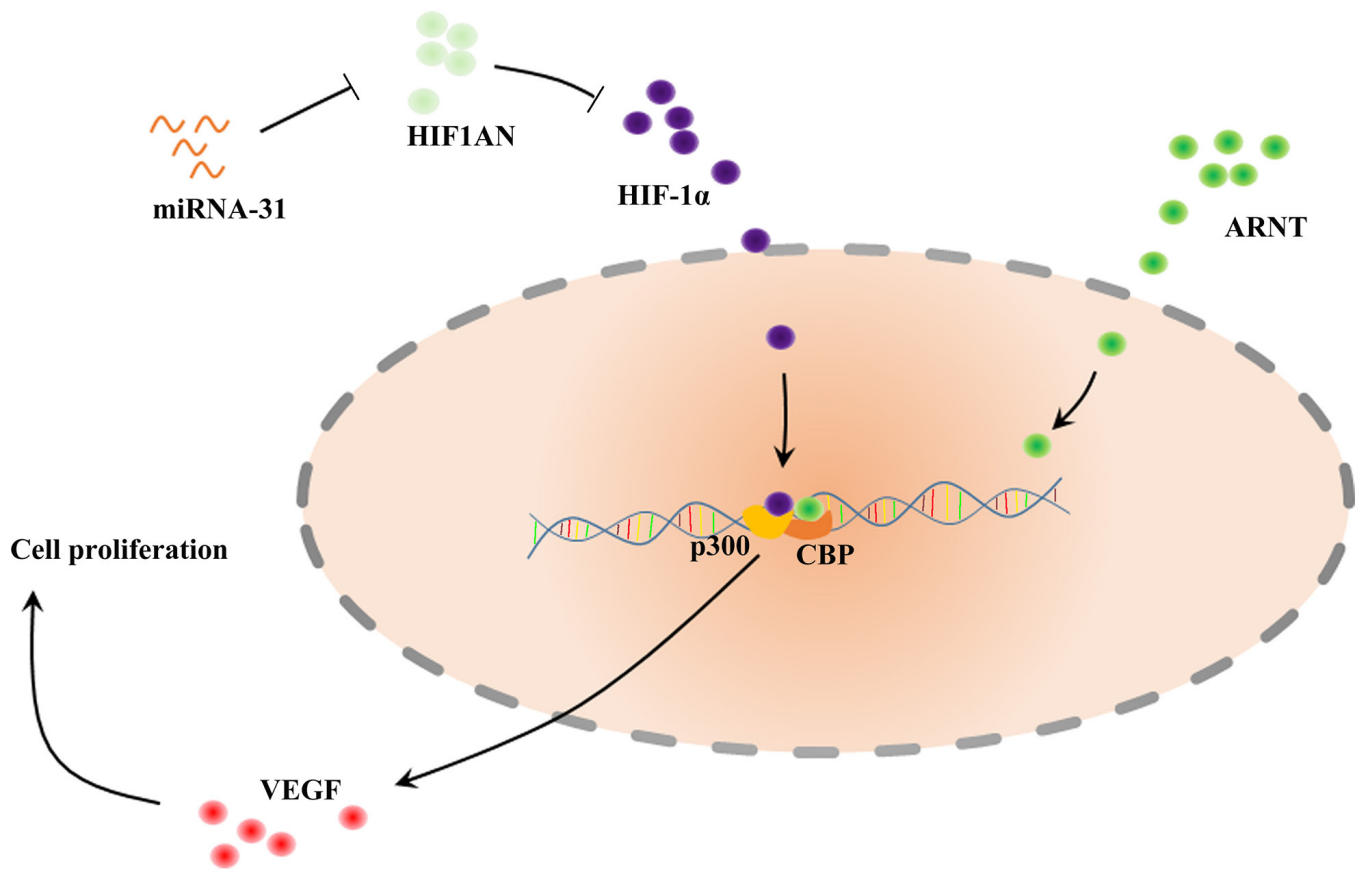
Schematic of the described interaction between miRNA-31, HIF1AN and VEGF HIF1α can bind ARNT (also known as HIF1β) to form heterodimer, which affects recruitment of the coactivators CBP and p300. HIF1AN, hypoxia inducible factor 1 alpha subunit inhibitor; HIF, Hypoxia-Inducible Factor; VEGF, Vascular Endothelial Growth Factor; ARNT, Aryl hydrocarbon Receptor Nuclear Translocator; CBP, CREB Binding Protein.

Here we confirmed that miRNA-31 was upregulated in keloid tissues and keloid-derived fibroblasts. We further found that *HIF1AN* was a target of miRNA-31. Previous studies demonstrated that HIF1AN, as an asparagine hydroxylase, has been shown to form a ternary complex with an E3 Ubiquitin ligase. HIF1AN could regulate the stability and activity of HIF-1α. Hydroxylation of HIF-1α by HIF1AN blocks the coactivators CREB binding protein (CBP) and the 300-kilodalton coactivator protein (p300), thus inhibites transcriptional activation (Figure 10) [26–28]. Chen and Peng *et al* suggested miRNA-31/HIF1AN nexus altered cell proliferation, migration, and invasion in colorectal cancer cell lines and contributed to keratinocyte differentiation [24, 29]. In this study, through loss-of-function strategies, we found that miRNA-31 could regulate cell proliferation, apoptosis and cell cycle progression of keloid-derived fibroblasts by targeting *HIF1AN*. Therefore, this miRNA-31/HIF1AN nexus in keloid scarring agrees with previous studies [24, 29].

HIF1AN can also form a complex with Mindbomb 1 (Mib1) and Mib2, which is an essential modulator of Notch signaling [30]. Moreover, Wang *et al.* found that HIF1AN may inhibit HIF-mediated transcription of *GLUT1* and *VEGF-A* [31]. The vascular defects caused by HIF1AN over-expression could be alleviated by regulation of VEGF-A, suggesting that HIF1AN functions as an anti-angiogenic factor during fibrosis development [32, 33]. Our results demonstrated that VEGF participated in regulation process of miRNA-31/HIF1AN on biological function of fibroblasts. This suggests the role of VEGF as a crucial growth factor in keloid formation. Based on these results, our study suggests that upregulation of miRNA-31 inhibits *HIF1AN* expression and activates VEGF in keloid scarring. We consider that miRNA-31/HIF1AN/VEGF pathway may play a critical role in the process of keloid. However, the functional role of miRNA-31 *in vivo* requires further investigation.

Taken together, our findings indicate that miRNA-31 can induce the cell proliferation of keloid-derived fibroblasts by down-regulating *HIF1AN*. Manipulation of miRNA-31 may represent a novel therapeutic strategy for treating the keloid scarring.

## MATERIALS AND METHODS

### Studies selection and integrated bioinformatics analysis

We retrieved studies that explored miRNA expression profiling of keloid and scar. The systematic search strategy was carried out using MEDLINE and ArrayExpress. The keywords and free text words used for this research were: (miRNA OR microRNA) AND profil* AND (keloid OR scar). We also performed hand searches of articles on this topic. Studies were excluded in our study if they didn’t use microRNA microarray. The study using only cell lines and serum was also excluded. All database searches were restricted to human subjects without language limitation.

Differential expression miRNA between keloid/scar and controls were extracted from the included studies. Then we tried to standardize the miRNA names according to miRBase database (Release 21, http://www.mirbase.org/). Cytoscape software platform was used to integrate these miRNAs of the included studies and visualize the networks.

### Sample preparation, fibroblasts isolation and culture

We enrolled keloid patients and healthy controls at the Second Affiliated Hospital of Kunming Medical University between October, 2014 and May, 2015. The detailed medical history and clinical features of the patients with keloid were obtained from the medical records. All patients had not received topical and systemic therapy for at least 2 months before performing a skin biopsy. Skin biopsy was temporarily stored in the RNA later (QIAGEN Group) before RNA was extracted. Written consent was gathered from all participants before the study was performed. The study was approved by the research ethics committee of the Second Affiliated Hospital of Kunming Medical University.

Keloid skin tissue samples (5 total) and normal skin tissue samples (5 total) were excised, cut into 1 mm^3^ pieces and then digested with 0.25% trypsin at 37°C. After tissue pieces were washed and mechanically dissociated, the cells were filtered, centrifuged, and resuspended in complete medium and cultured on dishes in a 37°C, 5% CO_2_ tissue-culture incubator.

### RNA isolation

Skin biopsies were ground to a powder in liquid nitrogen. Total RNA was extracted from skin biopsies and fibroblasts using mirVana® miRNA isolation kit (Ambion®, Carlsbad, CA, USA) following the manufacturer’s instructions. The quantity and quality of the extracted RNA were measured with Nanodrop-1000 spectrophotometer (Thermo Scientific, Wilmington, DE, USA) and Agilent 2100 Bioanalyzer (Agilent Technologies, Palo Alto, CA, USA). Finally the sample was stored at -80°C.

### Quantitative real-time RT-PCR (qRT-PCR)

miRNA-31 and *HIF1AN* were further quantitated by qRT-PCR using an ABI7500 Real-Time PCR System in tissues and fibroblasts. The cycling conditions were as follows: 95°C for 10 minutes, followed by 40 cycles of 95°C for 15 seconds, 60°C for 30 seconds, and 74°C for 5 seconds. U6 small nuclear RNA was used as an internal control. The threshold cycle (Ct) was defined as the fractional cycle number at which the fluorescence passed the fixed threshold. Each sample was measured in triplicate, and the relative amount of miRNAs to U6 was calculated. The relative expression of qPCR results was determined using comparative CT (2^-ΔΔCt^) method. Data analyses were performed via GraphPad Prism v6.00.

### Gene ontology analysis and pathway enrichment analysis

TargetScan, PicTar and miRanda were respectively used to predict putative target genes of miRNA-31. Cytoscape software was used for integrating miRNA-target genes interaction networks. To identify relevant biological pathways of miRNAs profiles, we performed gene ontology enrichment analysis and KEGG Pathway enrichment analysis using DAVID (Database for Annotation, Visualization and Integrated Discovery, http://david.abcc.ncifcif.gov/). They mainly focused on three terms which include biological process, molecular function and cellular component. The significant of both go and pathway enrichment analysis was set at the threshold p-value ≤ 0.05.

### Transfection of miRNA-31 mimic and miRNA-31 inhibitor

We obtained the sequence of the mature miRNA-31-5p from miRBase (http://www.mirbase.org/). The miRNA-31 mimic or miRNA-31 inhibitor and negative control (NC) were chemically synthesized by Ribobio Co. Ltd. (Guangdong, China). When keloid-derived fibroblasts grew to 50-70% confluence, miRNA-31 mimic or miRNA-31 inhibitor was transfected and incubated at 37 °C.

### Western blotting

The tissues and cultured cells were lysed and the protein was extracted using RIPA buffer (Solarbio, Beijing, China). The protein was quantified using a BCA kit (Sangon Biotech, Shanghai, China) according to the manufacturer’s instructions. Protein lysates were separated using 10% SDS-PAGE gel electrophoresis and transferred to PVDF membrane (Bio-Rad, Richmond, CA, USA). Antibodies used for Western blotting were: anti-HIF1AN (Sangon Biotech, Shanghai, China), anti-VEGF (Sangon Biotech, Shanghai, China), anti-HIF-1α (Sangon Biotech, Shanghai, China) and anti-Tubulin (Sangon Biotech, Shanghai, China). HRP-conjugated anti-mouse and anti-rabbit secondary antibodies (Santa Cruz, USA) were used. Finally, the membranes were treated with ECL reagents kit and exposed to X-ray film to detect the protein bands. Relative expression of relevant proteins were quantified and normalized to protein Tubulin.

### Cell counting Kit-8 assay

Cell proliferation activity was detected using CCK-8 assay (Solarbio, Beijing, China). In brief, fibroblasts which transfected with miRNA-31 inhibitor, *HIF1AN* siRNA and normal keloid-derived fibroblasts were seeded at 5×10^3^ cells per well in 96-well plates for triplicate. At 0 h, 24 h, 48 h, 72 h, and 96h, 10 uL of CCK-8 solution mixed with 100uL of DMEM was added into each well. After 0.5 hours incubation, absorbance was measured at 450 nm.

### EdU assay

Keloid-derived fibroblasts which transfected with miRNA-31 inhibitor, *HIF1AN* siRNA and normal fibroblasts were seeded at 96-well plates 1×10^4^ cells per well and incube 24 hours. Cells were cultured and EdU assay was performed according to the instruction. Photographs of the cells were captured with a fluorescent microscope equipped with a CCD camera (Nikon ECLIPSE Ti), and captured images were processed and analysed with Image-pro Plus software. S-phase cells were randomly selected from a single captured field, and the ratio of cell proliferation was calculated from five different fields.

### Cell apoptosis and cell cycle

Fibroblasts were collected and washed twice with PBS. Then cells were suspended in 200 μL of binding buffer. 5 μL of Annexin V-fluorescein isothiocyanate and 5 μL of propidium iodide (PI) were added for cell apoptosis assay. 500 μL of PBS containing 50 μg/mL PI and 50 μg/mL RNA enzyme were added for cell cycle assay. Then the cell suspensions were incubated in dark at room temperature for more than 30 min. The expression of the fluorescent was measured using the flow cytometer.

### 3’-UTR luciferase reporter assay

The forward and reverse segment including miR-31-binding sites in 3’-untranslated region (3’-UTR) of *HIF1AN* was synthesized by Ribobio Co. Ltd. (Guangdong, China). Then segment was inserted into the XbaI and FseI sites of pGL3 control vector and generated wild-type (WT) and mutant (MUT) pGL3-control-HIF1AN. Keloid-derived fibroblasts were seeded and co-transfected with the above constructs and miRNA-31 expression vector, miRNA-31 inhibitor, control vector or negative control. After 48 h, fibroblasts were harvested and luciferase activity was analyzed using the Dual-Luciferase Reporter Assay System (Biotek synergy, USA).

### Statistical analysis

All analyses were carried out with SPSS software (version 17.0, USA). Statistical analysis was carried out with a Student’t test for the comparison of two groups and with a Fisher’s exact probability test for the analysis of frequency. The relevant data were expressed as the mean ± SEM of at least three separate experiments. In this study, the p values 0.05 were considered significant.

## References

[1] Mari W, Alsabri SG, Tabal N, Younes S, Sherif A, Simman R (2015). Novel Insights on Understanding of Keloid Scar: Article Review. J Am Coll Clin Wound Spec.

[2] Jones ME, Hardy C, Ridgway J (2016). Keloid Management: A Retrospective Case Review on a New Approach Using Surgical Excision, Platelet-Rich Plasma, and In-office Superficial Photon X-ray Radiation Therapy. Adv Skin Wound Care.

[3] Jones ME, McLane J, Adenegan R, Lee J, Ganzer CA (2017). Advancing Keloid Treatment: A Novel Multimodal Approach to Ear Keloids. Dermatol Surg.

[4] Tirgan MH (2016). Neck keloids: evaluation of risk factors and recommendation for keloid staging system. F1000Res.

[5] He Y, Deng Z, Alghamdi M, Lu L, Fear MW, He L (2017). From genetics to epigenetics: new insights into keloid scarring. Cell Prolif.

[6] Ma L. MicroRNA and Metastasis. Adv Cancer Res. 2016; 132: 165–207. https://doi.org/10.1016/bs.acr.2016.07.004 10.1016/bs.acr.2016.07.004 27613133

[7] Liu Y, Wang X, Yang D, Xiao Z, Chen X (2014). MicroRNA-21 affects proliferation and apoptosis by regulating expression of PTEN in human keloid fibroblasts. Plast Reconstr Surg.

[8] Yu X, Li Z, Chan MT, Wu WK (2015). microRNA deregulation in keloids: an opportunity for clinical intervention?. Cell Prolif.

[9] Liu Y, Yang D, Xiao Z, Zhang M (2012). miRNA expression profiles in keloid tissue and corresponding normal skin tissue. Aesthetic Plast Surg.

[10] Zhang GY, Wu LC, Liao T, Chen GC, Chen YH, Zhao YX, Chen SY, Wang AY, Lin K, Lin DM, Yang JQ, Gao WY, Li QF (2016). A novel regulatory function for miR-29a in keloid fibrogenesis. Clin Exp Dermatol.

[11] Luan Y, Liu Y, Liu C, Lin Q, He F, Dong X, Xiao Z (2016). Serum miRNAs Signature Plays an Important Role in Keloid Disease. Curr Mol Med.

[12] Kashiyama K, Mitsutake N, Matsuse M, Ogi T, Saenko VA, Ujifuku K, Utani A, Hirano A, Yamashita S (2012). miR-196a downregulation increases the expression of type I and III collagens in keloid fibroblasts. J Invest Dermatol.

[13] Li C, Bai Y, Liu H, Zuo X, Yao H, Xu Y, Cao M (2013). Comparative study of microRNA profiling in keloid fibroblast and annotation of differential expressed microRNAs. Acta Biochim Biophys Sin (Shanghai).

[14] Wu ZY, Lu L, Liang J, Guo XR, Zhang PH, Luo SJ (2014). Keloid microRNA expression analysis and the influence of miR-199a-5p on the proliferation of keloid fibroblasts. Genet Mol Res.

[15] Li P, He QY, Luo CQ (2014). Overexpression of miR-200b inhibits the cell proliferation and promotes apoptosis of human hypertrophic scar fibroblasts *in vitro*. J Dermatol.

[16] Guo L, Xu K, Yan H, Feng H, Chai L, Xu G (2016). Expression Profile of Long Noncoding RNAs in Human Earlobe Keloids: A Microarray Analysis. Biomed Res Int.

[17] Guo XR, Liang J, Huang RL, Lu L, Jin YD, Luo SJ (2012). ZY. W. Differential expression of microRNAs in human keloids. Zhongguo Zuzhi Gongcheng Yanjiu.

[18] Rang Z, Wang ZY, Pang QY, Wang YW, Yang G, Cui F (2016). MiR-181a Targets PHLPP2 to Augment AKT Signaling and Regulate Proliferation and Apoptosis in Human Keloid Fibroblasts. Cell Physiol Biochem.

[19] An G, Liang S, Sheng C, Liu Y, Yao W (2017). Upregulation of microRNA-205 suppresses vascular endothelial growth factor expression-mediated PI3K/Akt signaling transduction in human keloid fibroblasts. Exp Biol Med (Maywood).

[20] Wu ZY, Lu L, Guo XR, Zhang PH (2013). [Identification of differently expressed microRNAs in keloid and pilot study on biological function of miR-199a-5p]. Zhonghua Zheng Xing Wai Ke Za Zhi.

[21] Yan S, Xu Z, Lou F, Zhang L, Ke F, Bai J, Liu Z, Liu J, Wang H, Zhu H, Sun Y, Cai W, Gao Y (2015). NF-kappaB-induced microRNA-31 promotes epidermal hyperplasia by repressing protein phosphatase 6 in psoriasis. Nat Commun.

[22] Xu N, Meisgen F, Butler LM, Han G, Wang XJ, Soderberg-Naucler C, Stahle M, Pivarcsi A, Sonkoly E (2013). MicroRNA-31 is overexpressed in psoriasis and modulates inflammatory cytokine and chemokine production in keratinocytes via targeting serine/threonine kinase 40. J Immunol.

[23] Wang A, Landen NX, Meisgen F, Lohcharoenkal W, Stahle M, Sonkoly E, Pivarcsi A (2014). MicroRNA-31 is overexpressed in cutaneous squamous cell carcinoma and regulates cell motility and colony formation ability of tumor cells. PLoS One.

[24] Peng H, Kaplan N, Hamanaka RB, Katsnelson J, Blatt H, Yang W, Hao L, Bryar PJ, Johnson RS, Getsios S, Chandel NS, Lavker RM (2012). microRNA-31/factor-inhibiting hypoxia-inducible factor 1 nexus regulates keratinocyte differentiation. Proc Natl Acad Sci U S A.

[25] Asangani IA, Harms PW, Dodson L, Pandhi M, Kunju LP, Maher CA, Fullen DR, Johnson TM, Giordano TJ, Palanisamy N, Chinnaiyan AM (2012). Genetic and epigenetic loss of microRNA-31 leads to feed-forward expression of EZH2 in melanoma. Oncotarget.

[26] Shin DH, Li SH, Chun YS, Huang LE, Kim MS, Park JW (2008). CITED2 mediates the paradoxical responses of HIF-1alpha to proteasome inhibition. Oncogene.

[27] Ruas JL, Poellinger L, Pereira T (2005). Role of CBP in regulating HIF-1-mediated activation of transcription. J Cell Sci.

[28] Pawlus MR, Wang L, Ware K, Hu CJ (2012). Upstream stimulatory factor 2 and hypoxia-inducible factor 2alpha (HIF2alpha) cooperatively activate HIF2 target genes during hypoxia. Mol Cell Biol.

[29] Chen T, Yao LQ, Shi Q, Ren Z, Ye LC, Xu JM, Zhou PH, Zhong YS (2014). MicroRNA-31 contributes to colorectal cancer development by targeting factor inhibiting HIF-1alpha (FIH-1). Cancer Biol Ther.

[30] Tseng LC, Zhang C, Cheng CM, Xu H, Hsu CH, Jiang YJ (2014). New classes of mind bomb-interacting proteins identified from yeast two-hybrid screens. PLoS One.

[31] Wang E, Zhang C, Polavaram N, Liu F, Wu G, Schroeder MA, Lau JS, Mukhopadhyay D, Jiang SW, O’Neill BP, Datta K, Li J (2014). The role of factor inhibiting HIF (FIH-1) in inhibiting HIF-1 transcriptional activity in glioblastoma multiforme. PLoS One.

[32] Hu J, Chen C, Liu Q, Liu B, Song C, Zhu S, Wu C, Liu S, Yu H, Yao D, Kang J, Zhu L (2015). The role of the miR-31/FIH1 pathway in TGF-beta-induced liver fibrosis. Clin Sci (Lond).

[33] Kiriakidis S, Henze AT, Kruszynska-Ziaja I, Skobridis K, Theodorou V, Paleolog EM, Mazzone M (2015). Factor-inhibiting HIF-1 (FIH-1) is required for human vascular endothelial cell survival. FASEB J.

